# Morphological characterization using scanning electron microscopy of fly artifacts deposited by *Calliphora vomitoria* (Diptera: Calliphoridae) on household materials

**DOI:** 10.1007/s00414-021-02634-8

**Published:** 2021-07-01

**Authors:** Guido Pelletti, Desiree Martini, Laura Ingrà, Maria Carla Mazzotti, Arianna Giorgetti, Mirella Falconi, Paolo Fais

**Affiliations:** 1 Department of Medical and Surgical Sciences, Unit of Legal Medicine, University of Bologna, Via Irnerio 49, 40126 Bologna, Italy; 2 Department of Biomedical and Neuromotor Sciences, University of Bologna, Bologna, Italy

**Keywords:** Forensic pathology, Crime scene, Bloodstain pattern analysis, Fly artifacts, Scanning electron microscopy

## Abstract

Insects found at a crime scene can produce traces referred to as fly artifacts (FA) due to their movement over the corpse and the manner in which they feed upon it. These can be detrimental for carrying out criminal investigations. Confusing a FA with a genuine bloodspot can lead to misinterpretations, also taking into consideration that FA may contain a human DNA profile. The aim of the present study was to employ scanning electron microscopy (SEM) for the analysis of FA produced by *Calliphora vomitoria* on hard surfaces and fabrics that are commonly present at crime scenes. FA and control bloodstains were produced under experimental conditions on metal, glass, plaster, cotton, and polyester. After macroscopic analysis, FA were examined at standard low (20–40 ×), medium low (300–600 ×), and high ultrastructural (1200 ×) magnification through a SEM Stereoscan 360, Leica, Cambridge. SEM analysis enabled the identification of distinctive features of FA on hard surfaces, namely, amorphous crystals, micro-crystals with a morphology similar to those of uric or micro-crystals with a comparable morphology to cholesterol, absent in controls. Moreover, red blood cells (RBC) were absent in FA but were always present in controls. On cotton, for both FA and controls, the drop was almost completely absorbed and thus indistinguishable from the underlying fabric texture. On polyester, FA showed amorphous/crystal-like deposits and no RBC, as observed on hard surfaces, except for those showing a completely flat surface. SEM analysis appeared to be suitable for differential diagnosis between FA and genuine bloodstains on hard surfaces, although the results may be inconclusive on tested fabrics.

## Introduction

Insects found at a crime scene can be of use to many types of forensic investigation. They can provide information about time since death, season of death, the primary crime scene, movement or concealment of the remains following death, specific sites of trauma on the body, use of drugs, and victim identification when the body is removed from the initial decomposition site [1–3]. However, the presence of insects does not always aid investigations and may even prove counterproductive by reason of their movement over the corpse and the manner in which they feed on it. Flies in the Calliphoridae and Sarcophagidae families land on the corpse or on biological fluids, walking across the surfaces and feeding. Foraging activity is known to cause the most problematic alteration of the death scene, as insects can create unique stains or intermix fly artifacts with bloodstains and other human body fluids as a result of their digestive process [4]. Insects can also create transfer patterns with the tarsi or the abdomen or leave impressions after passing through the fluids [5]. Confusing FA with genuine bloodspots can lead to misinterpretations, also considering that FA may contain human DNA profile [6].

Certain macroscopic key features may help an investigator to distinguish fly artifacts from blood spots [7, 8]. However due to the wide range of shapes, colors, and sizes and the potentially high number of FA found at crime scenes, there is a strong probability that FA can appear very similar to genuine drops of blood [5]. In such cases, the visual morphologic approach should be integrated with a confirmatory technique for identification [9].

A recent study from our group showed that scanning electron microscopy (SEM) allows the visualization of ultrastructural morphological differences between FA deposited by *Sarcophaga carnaria* and blood controls on different types of paper — a promising tool for performing a differential diagnosis between FA and bloodstains [10].

However, the main problem associated with visual or ultrastructural stain morphology is that baseline data on fly stains deposited on materials typical of crime scenes, such as fabrics, metal, glass, or plaster is still scarce [11]. Moreover, the morphologic features of FA related to the activity of a fly may differ from the FA produced from other fly species [12].

The aim of the present study was to employ SEM for the analysis of FA produced by *Calliphora vomitoria* (*C. vomitoria*), on five different surfaces that can be commonly present in a crime scene, namely, metal, plaster, glass, cotton, and polyester, in order to obtain further information on the ultrastructural distinction between FA and genuine bloodstains.

## Materials and methods

### Scene and experiment

#### Scene

One hundred adults of *C. vomitoria* were placed in a scaled-down room analog, referred to hereinafter as the ‘‘fly box.” The fly box was 0.12 m^3^ (1 × 0.3 × 0.4 m) with five wooden walls and one transparent wall to allow observation. Five different household materials were attached to the fly box walls: glass, plaster, metal, cotton, and polyester. Fifty mL of fresh human blood with EDTA were placed in a 0.008 m^3^ box on the floor of the fly box and were used as a blood reservoir for blowflies.

#### Experiment

The blowflies were placed in the fly box for 48 h, and, after that period, the fly box was opened, and the household materials covering the walls were removed. Twenty-five FA, five for each type of material, were sampled and analyzed macroscopically and via SEM.

#### Control sample

The scene was re-arranged as previously described, but in this case, the fresh human blood placed in a 0.008 m^3^ box on the floor of the box was used to create a parent stain [13]. The parent bloodstain was shot through a cylindrical plastic stick until bloodstains were produced on the walls of the box. Five bloodstains with a diameter lower than 0.3 cm were randomly sampled from each type of surface and were used as controls.

### Experiment with defrosted blood

The absence of red blood cells in FA, which were always present in bloodstains, was one of the distinctive features observed in the previous study. In forensic casework, when the corpse is removed after longer PMI, it may be necessary to distinguish between spots of hemolyzed blood and FA produced from this source. In order to test the morphology of FA produced from hemolyzed blood, the *scene* and the *experiment* were repeated using fresh defrosted human blood taken from a living donor and preserved at − 20º C for 1 day, as a blood reservoir for blowflies.

### Analysis of spots

Aiming at dividing the FA obtained in the experiment in categories based on gross macroscopic features, all spots were photographed with an Olympus E-520 camera equipped with an Olympus Zuiko Digital 35 mm 1:35 Macro lens. Color, surface, shape of the body, edges, and tail were observed and described for each spot.

After visual analysis, all the spots were prepared for SEM analysis as follows: from each type of material, a little square sample (1.2 cm side) surrounding blood drops or FA was withdrawn. All samples were mounted on a suitable gold–palladium-coated stub with carbon substrate. Then the samples were examined (analyzed) at standard low (20–40 ×), medium low (300–600 ×), and high ultrastructural (1200 ×) magnification through a SEM Stereoscan 360, Leica, Cambridge, with electron secondary probe at 15 kV, to appreciate not only shape, but morphological ultrastructural characteristics such as the appearance of the surface of the spot, the presence of deposits of foreign material, and the spot cell morphology.

## Results

**Fig. 1:**
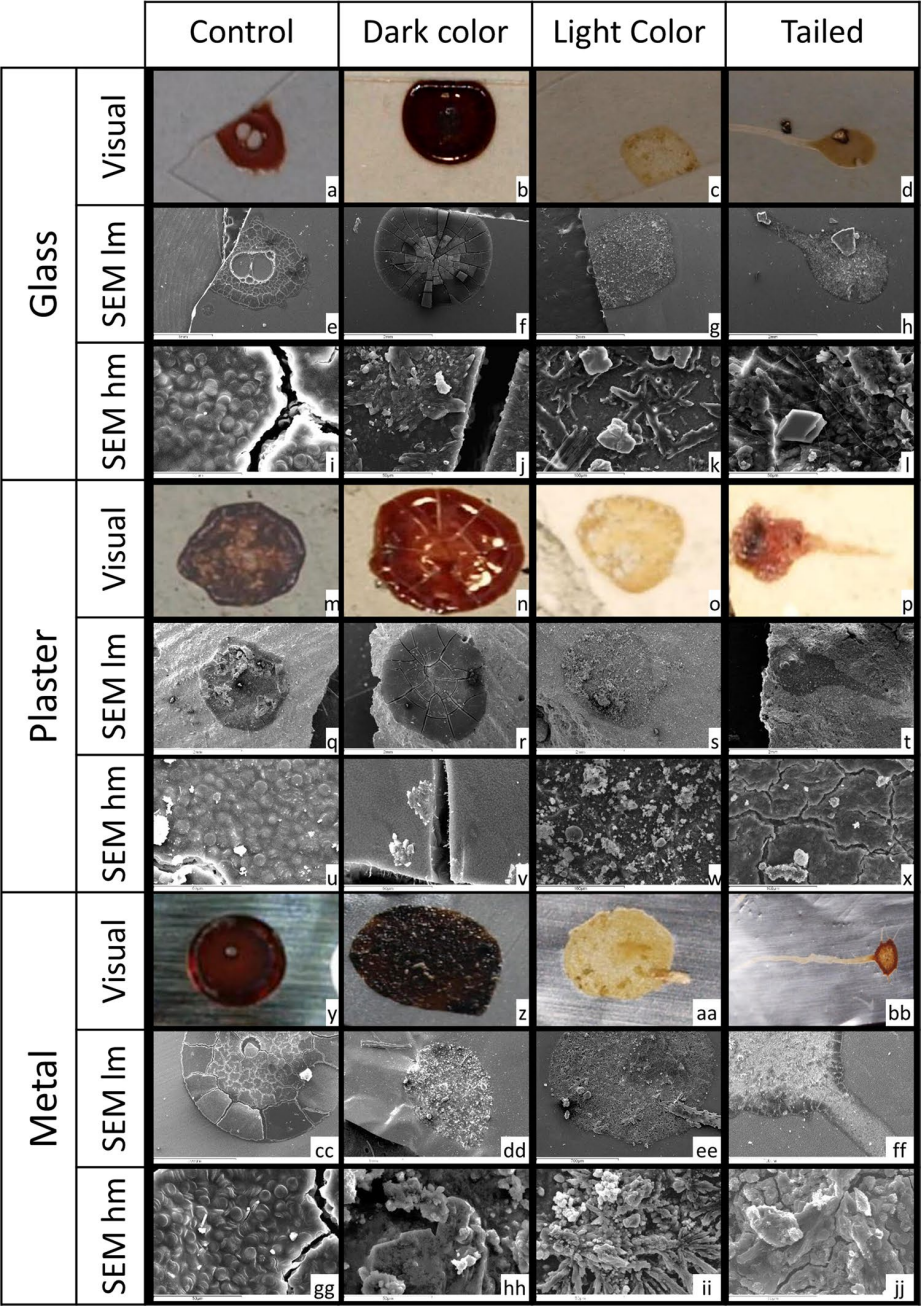
FA deposited on hard surfaces, namely, glass (a-l), plaster (m-x), and metal (j-jj). The figure reports the visual analysis, the low magnification SEM analysis (SEM lm), and the high magnification SEM analysis (SEM hm) of dark color FA, light color FA, tailed FA, and genuine bloodspots (control)

**Fig. 2:**
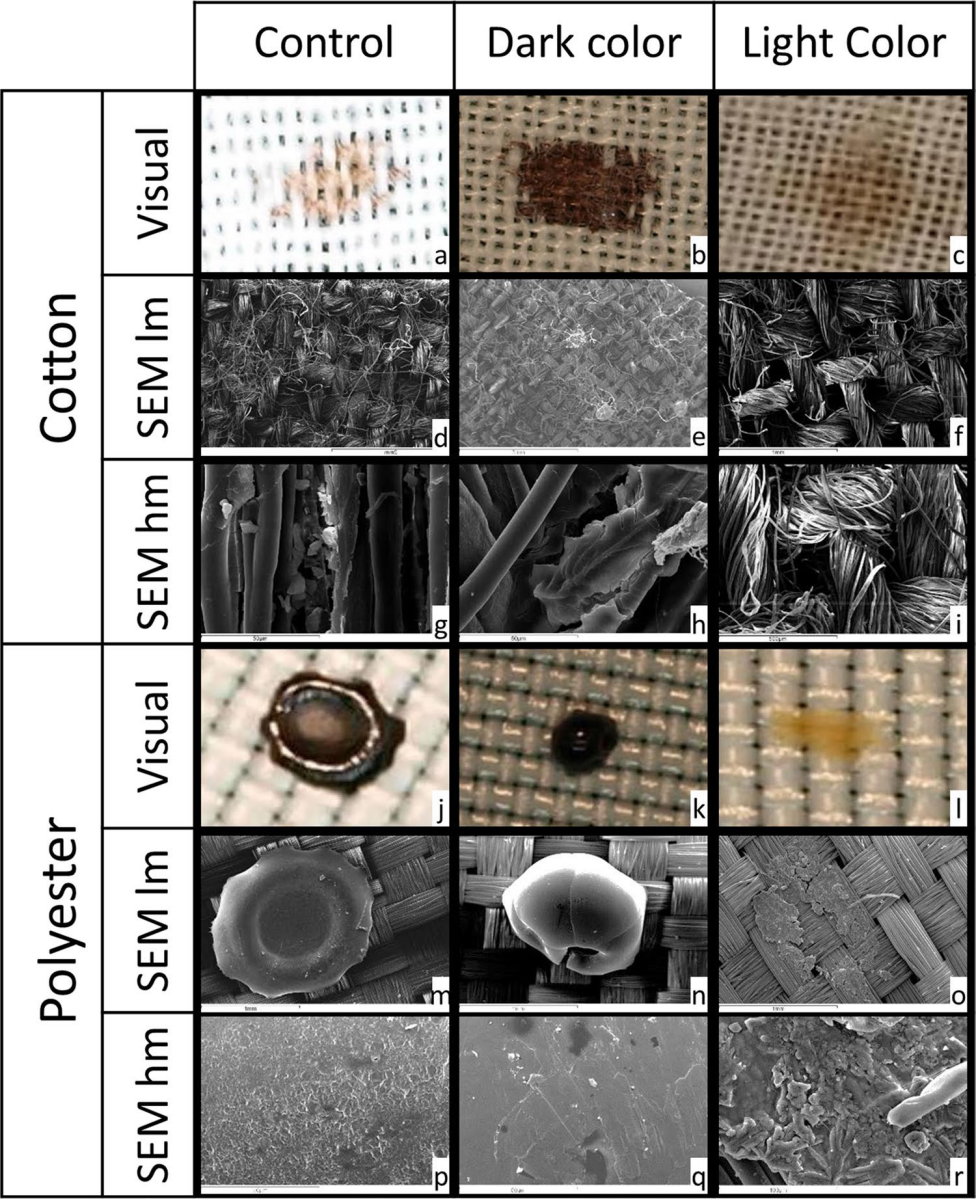
FA deposited on fabrics, namely, cotton (a-i) and polyester (j-r). The figure reports the visual analysis, the low magnification SEM analysis (SEM lm), and the high magnification SEM analysis (SEM hm) of dark color FA, light color FA, and genuine bloodspots (control)

The preliminary macroscopic analysis was aimed at identifying three categories of FA, based on gross visual features of the spots. FA on hard surfaces were divided into dark color FA (DFA), light color FA (LFA) and tailed FA (TFA). The results of visual analysis on hard surfaces are reported in Fig. 1 a–d (glass), m–p (plaster), and y–bb (metal). On cotton and polyester, FA with a tail clearly distinguishable from the body of the spot were absent. The results of visual analysis on fabrics are reported in Fig. 2 a–c (cotton) and j–l (polyester).

Visual features of FA on hard surfaces (glass, metal, plaster) are reported in Table 1, while visual features of FA on fabrics (cotton and polyester) are reported in Table 2.

**Table 1:** Gross visual features of FA on hard surfaces (glass, metal, polyester)

	Dark fly artifacts (DFA)	Light fly artifacts (LFA)	Tailed fly artifacts (TFA)	Controls (bloodspots)
Color	Red/brownish	Yellow/light brown	Red/brownish or yellow/light brown	Red/brownish
Shape of the body	Circular or elliptical			
Surface	Flat, cratered, or textured		Textured	Flat or cratered
Edges	Linear or slightly scalloped			
Tail	Absent or shorter than the body	Absent or shorter than the body	Longer than the body	Absent or shorter than the body

**Table 2:** Gross visual features of FA on fabrics (cotton and polyester)

	Dark fly artifacts (DFA)	Light fly artifacts (LFA)	Controls (bloodspots)
Color	Red/brownish	Yellow/light brown	Red/brownish
Shape of the body	Circular or elliptical	Circular, elliptical, or linear	Circular or elliptical
Surface (cotton)	Reproduced the texture of the underlying cotton fabric		
Surface (polyester)	Flat or cratered	Flat or textured	Flat or cratered
Edges (cotton)	Reproduced the underlying texture of the fabric		
Edges (polyester)	Linear, sometimes slightly scalloped		
Tail	Absent or shorter than the body		

### SEM analysis on hard surfaces

The results of SEM analysis on hard surfaces are reported in Fig. 1 e–l (glass), q–x (plaster), and cc–jj (metal).

FA showed on the surface of the small stain deposits, absent on the surface of control samples. Deposits on the surface of FA consisted of amorphous crystals, micro-crystals with morphology similar to those of uric, or micro-crystals with morphology similar to those of cholesterol.

Red blood cells (RBC) were absent in FA, while the surface of the controls was paved by RBC appreciable as biconcave discs, maintaining their own shape and appearing stacked in “rouleaux” due to drying fixation.

FA produced from defrosted hemolyzed blood showed crystals structurally similar to those previously observed. As expected, RBC were absent both in FA and in controls.

### SEM analysis on fabrics

The results of SEM analysis on fabrics are reported in Fig. 2 d–i (cotton) and m–r (polyester).

On cotton, for both FA and controls, the drop was almost completely absorbed and thus indistinguishable from the underlying texture of fabrics. Deposits and RBC were not visible both on FA and on controls.

On polyester, the surface of DFA was completely flat and sometimes cratered and was not explorable with SEM. Deposits and RBC on the surface of DFA and controls were not visible. The surface of LFA was glomerular. Deposits on the surface of LFA consisted of amorphous crystals and micro-crystals with morphology similar to those of uric or cholesterol. RBC were absent in LFA.

FA produced from defrosted hemolyzed blood showed crystals structurally similar to those previously observed. As expected, RBC were absent both in FA and in controls.

## Discussion

FA can display an innumerable variety of shapes due to the nature and number of variables that contribute to their formation, such as the different behaviors of fly species and the different deposition surfaces. Although studies have tried to produce FA under different experimental conditions on many surfaces, no complete consensus of physical attributes of FA has been reported [3, 8, 11, 14].

Durdle et al. [7] reported some specific features that improved the identification of FA at the crime scene based on their visual morphology. Following a rigorous scientific visual approach, most pitfalls related to classification can be avoided, especially when multiple stains are present and consequently specific features can be compared between different spots, such as the presence of a parent stain, the directionality, the color or shade of colors, the impact angle, and size [3, 15]. However, some types of FA, such as isolated dark red spots, are also difficult to distinguish from a bloodstain for educated and experienced pathologists and criminalists [15]. Moreover, experts in this distinction do not always attend crime scenes [14]. A confirmatory assay for the identification of FA is needed, especially in uncertain scenarios, where spots are of indeterminate origin also after a careful visual analysis.

The present study was developed to analyze FA produced by *C. vomitoria* on different surfaces that are commonly part of a crime scene. In particular, the aim of our investigation was to test the potential utility of SEM for distinguishing bloodstains from FA.

The preliminary macroscopic analysis was carried out to cluster FA into categories, namely, DFA, LFA, and TFA, on hard surfaces and DFA and LFA on fabrics. The absence of TFA on fabrics may be related to the particular behavior of stains on the array of textiles. In point of fact, when found on clothing apparel, the appearance of stains depends on fabric absorbency and texture, fabric construction (e.g., yarn size, twist level, fabric weight), and wear condition [16–19], as well as characteristics of the fluid itself, such as volume and composition [20].

On hard surfaces, at ultrastructural SEM analysis, a distinctive feature of FA was the presence at high magnification of amorphous crystals and/or micro-crystals with morphology similar to those of uric, which have been described in defecatory FA [4, 21], or cholesterol. Moreover, FA did not present RBC, while on controls, the surface of the stain was paved by RBC. These results were in agreement with the features observed on FA deposited by *S. carnaria* on paper [10].

In order to test the hypothesis of a correlation of the amorphous crystals and/or micro-crystal deposits with RBC degradation, the experiment was repeated analyzing FA produced by flies from defrosted hemolyzed blood. Furthermore, the presence of deposits on FA produced from hemolyzed blood support the hypothesis that deposits may be produced during the digestive activity of flies.

In forensic casework, for bloodstains that could originate from hemolyzed blood when the corpse is moved after longer PMI, the absence of RBC on the spot could lead to erroneous interpretation. To be correctly qualitatively identified as a FA, the stain should present the deposits described in the present study that were always present in all FA deposited on hard surfaces.

On fabrics, SEM analysis was cumbersome. In fact, on cotton, the complete absorption of the stain, which was appreciable also macroscopically, confounded the ultrastructural analysis. On both controls and FA, it was only possible to observe non-specific residues of biological material that did not display the features of RBC, neither of the amorphous nor crystal-like material.

On polyester, the surface of DFA and controls was completely flat. Consequently, the beam of accelerated electrons could not penetrate the surface of the bloodspot because of the absence of superficial cracks and it was therefore impossible to appreciate RBC neither to amorphous/crystal-like material and to make a differential diagnosis.

The surface of LFA displayed uric/cholesterol-like and amorphous deposits; RBC were absent, as observed on hard surfaces, allowing differential diagnosis.

**Fig. 3:**
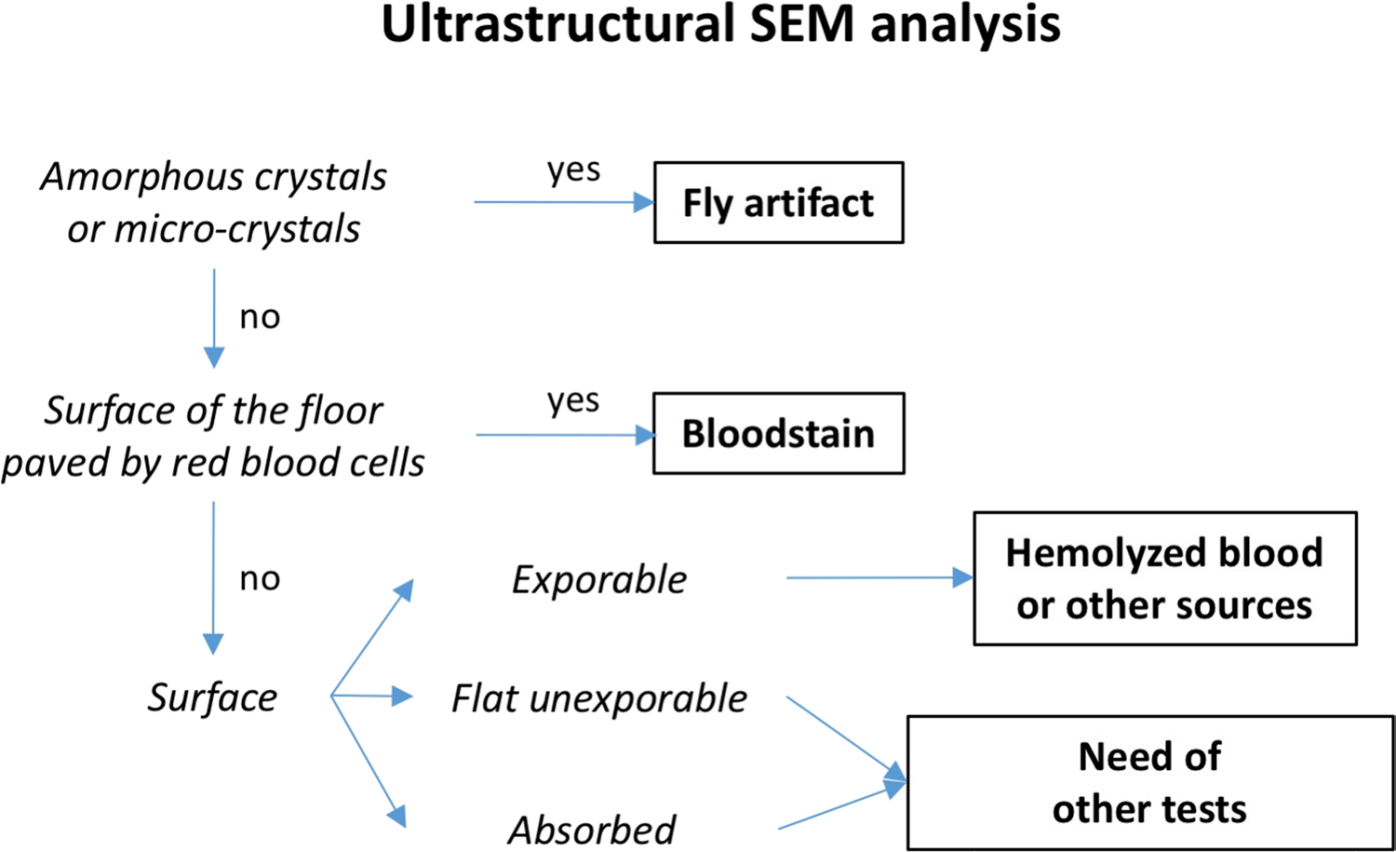
Flow chart depicting how a fly artifact can be distinguished from a blood spot using SEM ultrastructural analysis

Based on the results obtained from the present study, we report in Fig. 3 a flow chart of the potential application of SEM ultrastructural analysis. In some cases, the origin of the stain can remain undetermined even after a combined macroscopic ultrastructural approach, such as in the case of dark-colored stains with a flat surface that cannot be explored by SEM, like those we observed on polyester, or stains absorbed by the deposition surface, like those we observed on cotton. In these cases, other non-morphological techniques could be used, such as the recently developed immunodetection with polyclonal antiserum [11, 12, 14, 22]. Even if validation studies are needed on other experimental settings, SEM analysis confirmed to be a promising tool for distinctions of FA from true human bloodstains.

The presented method has some unavoidable limitations, due to the technique of analysis and the type of instrument used. First, SEM is not readily available to all forensic medicine services, being an expensive technique that sees few and specific forensic applications. In any case, the forensic experts who intervene during the crime scene investigation must be aware of the possibility of using this technique in the case of macroscopically indistinguishable spots, as an integrative technique to those already proposed in the literature. Furthermore, the preparation of the sample involves an irreversible modification of the spot, which cannot be used for further analysis. Therefore, SEM analysis must be performed as a last resort, after resorting to other more conservative approaches. Another issue is related to the phase of sampling, since for the analysis, it is necessary to sample not only the spot, but also the underlying substrate, such as the flash of a window or a part of plaster. This implies an irreversible modification of the crime scene. For this reason, we recommend the sampling for SEM analysis following an accurate high-resolution photographic collection of the spots, according to shared procedures [3].

## Conclusions

As previously observed on FA deposited by *S. carnaria* on different types of paper, SEM analysis, through the investigation of surface deposits and RBC, was confirmed as suitable for differential diagnosis on hard surfaces such as metal, glass, and plaster. On fabrics, namely, cotton and polyester, SEM analysis may be inconclusive. When it is necessary to identify the origin of an ambiguous stain and insect activity is suspected, a multidisciplinary approach to bloodstain pattern analysis is strongly suggested, including SEM analysis.
